# The effect of face-to-face versus online learning on student performance in anatomy: an observational study using a causal inference approach

**DOI:** 10.1007/s44217-022-00027-6

**Published:** 2023-01-04

**Authors:** Joanna Diong, Hopin Lee, Darren Reed

**Affiliations:** 1 School of Medical Sciences, Faculty of Medicine and Health, The University of Sydney, Camperdown, Australia; 2 Nuffield Department of Orthopaedics, Rheumatology and Musculoskeletal Sciences, University of Oxford, Oxford, UK

**Keywords:** Anatomy, Learning, Education, Distance, Education, Medical, Continuing

## Abstract

**Introduction:**

This study aimed to estimate the causal effect of face-to-face learning on student performance in anatomy, compared to online learning, by analysing examination marks under a causal structure.

**Methods:**

We specified a causal graph to indicate how the mode of learning affected student performance. We sampled purposively to obtain end-semester examination marks of undergraduate and postgraduate students who learned using face-to-face (pre-COVID, 2019) or online modes (post-COVID, 2020). The analysis was informed by the causal graph. Marks were compared using linear regression, and sensitivity analyses were conducted to assess if effects were robust to unmeasured confounding.

**Results:**

On average, face-to-face learning improved student performance in the end-semester examination in undergraduate students (gain of mean 8.3%, 95% CI 3.3 to 13.4%; E-value 2.77, lower limit of 95% CI 1.80) but lowered performance in postgraduate students (loss of 8.1%, 95% CI 3.6 to 12.6%; E-value 2.89, lower limit of 95% CI 1.88), compared to online learning.

**Discussion:**

Under the assumed causal graph, we found that compared to online learning, face-to-face learning improved student performance in the end-semester examination in undergraduate students, but worsened student performance in postgraduate students. These findings suggest that different modes of learning may suit different types of students. Importantly, this is the first attempt to estimate causal effects of the mode of learning on student performance under a causal structure. This approach makes our assumptions transparent, informs data analysis, and is recommended when using observational data to make causal inferences.

## Introduction

The study of anatomy is foundational to medical and paramedical degrees. The traditional approach to anatomy higher education usually involves face-to-face lectures and practical/tutorial classes using cadaver specimens, models, and other media. A key premise of this approach is that having students examine specimens ‘hands-on’ in three dimensions with guidance from teachers present in person is the gold standard of anatomy education in medicine and health [1].

Nevertheless, investigators have explored including online components with face-to-face anatomy education, or replacing face-to-face anatomy education with online components, even prior to the COVID-19 pandemic [2]. Online anatomy education can differ markedly from the gold standard of face-to-face learning by allowing content to be paused, replayed and viewed on demand [3, 4]. However, online education also reduces or removes students’ physical access to cadaver specimens, other media, and in-person interactions with teachers, which may affect cadaver-based identification and explanatory knowledge [5]. To what extent does face-to-face learning improve student performance in anatomy, compared to online learning?

When framed as above, this question seeks to identify the *causal* effect of face-to-face learning on student performance, compared to online learning, and has been investigated in a number of observational, non-randomised studies. Previous attempts to identify causal effects of face-to-face versus online learning on student performance in anatomy, using observational studies, report conflicting findings. For example, undergraduate occupational therapy students who learned in face-to-face gross anatomy laboratory classes achieved higher overall grades, self-perceived learning and satisfaction, than students who learned using online anatomy software [6]. However, face-to-face students also spent more time studying anatomy than online students, which could explain why they performed better [6]. In contrast, undergraduate dental students who learned in face-to-face classes achieved lower cumulative examination marks, than students who learned in online classes [7]. Lastly, graduate medical and speech pathology students made comparable knowledge gains regardless whether they learned in face-to-face lectures or in an online interactive course [8].

The key limitation in many observational studies is the lack of knowledge of the causal structure by which the mode of learning affects student performance. Without knowledge of the causal structure, it is not possible to ascertain if performance was compared between groups of students that are *exchangeable* (i.e. groups are balanced such that the same findings would be obtained regardless of which group was allocated the experimental or control condition). Consequently, it is not possible to identify the assumptions behind how learning affects performance, and findings may be subject to bias by confounding [9, 10] and thus conflict.

To address this limitation, this study aimed to estimate the causal effect of face-to-face compared to online learning on student performance in anatomy, by analysing examination marks in different cohorts of undergraduate and postgraduate students under a causal structure. We estimated the total effect of the mode of learning on student performance, and provide between-group mean differences and 95% CI.

## Methods

For context, early in 2020, greater metropolitan Sydney was placed under lockdown to mitigate the spread of the COVID-19 virus. All anatomy units rapidly transitioned from face-to-face to fully online learning in semester 1, week 4. In the remaining 9 of the 13 weeks of semester 1, all anatomy learning and all examinations were conducted online. All units and examinations in semester 2, 2020 were also conducted online.

We analysed observational data using a causal inference approach to investigate how the mode of learning affects student performance [9]. We purposively sampled cohorts of undergraduate and postgraduate students in anatomy at The University of Sydney who learned either by face-to-face or online modes, and compared examination marks between cohorts. The analysis of examination marks was informed by a causal structure. No formal sample size calculation was performed. However, given that data on examination marks are continuous and cohort sizes are large (historically, n = 60–80 students enrolled), a sample size of 60 per group would provide 80% power to detect a between-group standardised effect size of 0.5, with alpha = 0.05.

### Causal structure

**Fig. 1:**
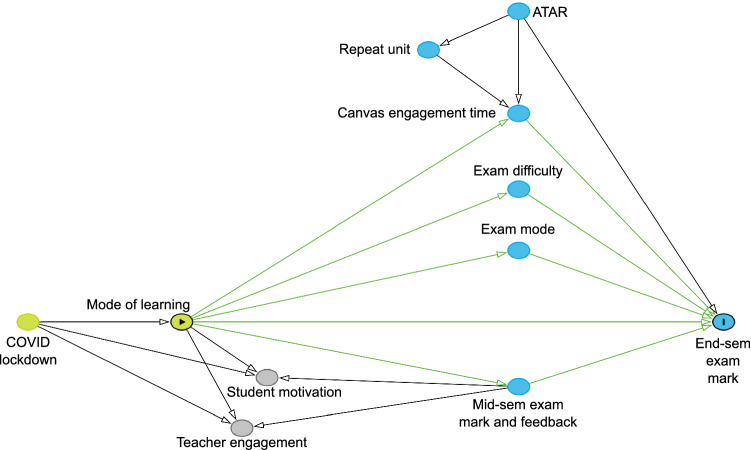
Causal directed acyclic graph of the plausible effect of the mode of learning (exposure) on student performance in the end-semester examination (outcome). Arrows indicate the direction of causation between nodes. The total effect of the mode of learning on the end-semester examination mark is shown (green arrows)

Specifying a theoretical causal structure in a directed acyclic graph to identify causal effects is increasingly used in observational studies, and is an explicit and transparent approach to represent the plausible mechanisms by which the mode of learning affected student performance in examinations [11, 12]. In our casual graph, we investigate how the mode of learning affected student performance in examinations (Fig. 1). We specified this graph based on evidence that suggests time spent studying anatomy [6], examination difficulty [13], examination mode [14], feedback [15, 16], and prior academic calibre [17] are plausible mechanisms by which the mode of learning affects student performance. The arrows indicate the direction of causation and do not cycle back on themselves. Under our proposed causal graph, the COVID lockdown caused students to learn under a certain mode, which affected their end-semester examination mark. Before the lockdown, students learned by face-to-face mode; during the lockdown, students learned by online mode.

Under the assumed causal graph, the total effect of the mode of learning on the end-semester examination mark was assumed to be mediated through the amount of time that students engaged with the anatomy content in the unit’s Canvas learning management system, the difficulty of the examination, the mode of the examination (i.e. face-to-face or online), and the mid-semester examination mark. Academically stronger students who achieved higher Australian Tertiary Admission Rank (ATAR) values may have been more conscientious and likely spent more time engaging with content in Canvas. Students with lower ATARs who repeated the unit may have spent more time engaging with the content in Canvas. Prior academic calibre likely also affected the end-semester examination mark achieved.

The COVID-19 lockdown may have affected student performance through mechanisms other than the mode of learning [18, 19], such as through student motivation to learn anatomy [20], and how teachers engaged with students [21]. If these alternative mechanisms prevail, the causal effect of the mode of learning on student performance cannot be identified. However, students and teachers likely used the mid-semester examination mark and feedback from that examination to mitigate or block the effects of the lockdown and the mode of learning on student motivation and teacher engagement. This is shown by the arrows from *mid-sem exam mark and feedback* pointing back to *student motivation* and *teacher engagement* nodes (Fig. 1). If this mitigation is complete, under the causal graph, there are no other plausible causal effects of student motivation and teacher engagement on the end-semester examination mark.

Thus, under the assumed causal graph, the total effect of the mode of learning on student performance can be assessed by comparing the difference in the end-semester marks alone (importantly, not the combined mid- and end-semester marks) between cohorts that learned by face-to-face versus online modes. No other adjustment for baseline confounders is necessary to estimate the total effect.

The causal graph was specified using the R package DAGitty (http://www.dagitty.net/) [22] to obtain the minimally-sufficient set of variables for adjustment, if any are needed, to determine the total effect. The causal graph was developed and revised by the investigator team, and refined in consultation with other members of the teaching team. This study was approved by the University of Sydney Human Research Ethics Committee (2021/125), in accordance with the ethical standards as laid down in the 1964 Declaration of Helsinki and its later amendments. The study protocol was pre-registered on the Open Science Foundation (https://osf.io/xhs83/).

### Participants

Functional musculoskeletal anatomy is taught to undergraduate and postgraduate students in health sciences at the School of Medical Sciences, The University of Sydney. Students enrolled in 2019 learned by face-to-face mode, while students enrolled in 2020 learned by online mode due to the COVID-19 imposed lockdown. Participants were included if they were undergraduate or postgraduate students enrolled in upper limb anatomy units in 2019 and 2020. We analysed examination marks data from these students.

Most undergraduate students were enrolled in Bachelor programs in physiotherapy, occupational therapy, exercise and sport science, exercise physiology, and diagnostic radiography. Some undergraduate students were enrolled in ‘non-standard’ professions (engineering, nursing, science, health science, advanced studies). We analysed data from students enrolled in semester 2 of 2019 and 2020.

All postgraduate students were enrolled in a Masters of occupational therapy. We analysed data from students enrolled in semester 1 of 2019 and 2020.

### Description of units

Both the undergraduate and postgraduate units consisted of modules that covered the bones, joints and ligaments, muscles, nerve supply, blood supply and surface anatomy of the upper limb, with an emphasis on function and human movement analysis. The units also covered introductory histology content, including osteology, arthrology, myology, generalised connective tissue, and bone tissue and growth. The postgraduate unit contained an additional module on the vertebral column. All modules in both units were spread over 13 weeks of the semester, and comprised of 2 h of lectures and 2 h of practical or tutorial classes each week. The time commitment for all lessons in face-to-face and online cohorts was identical.

For both units, content in the modules was taught as follows:

- Face-to-face (2019 cohorts): Students attended lectures in person. By default, all lectures were recorded and made available to students in Canvas. Students attended practical classes in anatomy laboratories. They learned the content using media such as cadaver specimens, bones, anatomical models, X-rays and surface anatomy photographs. The teacher:student ratio in practical classes was ~ 1:25.
- Online (2020 cohorts): All lectures were pre-recorded and made available in Canvas, or delivered live online using Zoom software. Students attended online tutorials using Zoom. They learned the content using images of cadaver specimens, bones, anatomical models, X-rays and surface anatomy, through learning activities in online resources. Student learning could occur in the tutorial group as a whole, or in smaller ‘breakout’ rooms. The teacher:student ratio was still ~ 1:25.

### Description of examinations

For both units, content from weeks 1–7 was assessed in the mid-semester examination, and content from the whole semester focusing on weeks 8–13 was assessed in the end-semester examination(s). All examinations used multiple choice questions to assess student understanding of structure and function in musculoskeletal anatomy. In general, the multiple choice questions used were as reliable and valid as free-text response questions [23].

In the undergraduate unit,

- Face-to-face: Students were examined in a mid-semester practical examination (30% of total mark), and end-semester practical (30%) and theory (40%) examinations. Practical examinations were conducted in the anatomy laboratories, and examined students using media such as cadaver specimens, bones, anatomical models, X-rays and surface anatomy photographs. The end-semester theory examination did not include media, and examined content from the whole semester. All face-to-face examinations were paper-based, and students were supervised by human invigilators.
- Online: Students were examined in mid-semester (40% of total mark) and end-semester (60%) online examinations. Both examinations were conducted in Canvas. Many multiple choice questions in the online examinations included images of cadaver specimens, bones, anatomical models, X-rays and surface anatomy. Students were supervised using ProctorU Review + artificial intelligence software.

In the postgraduate unit, the introductory histology content was examined in an online quiz (5% of total mark; week 3) in both face-to-face and online student cohorts. This content was not examined again in the mid- or end-semester examinations. Since the introductory histology content was examined under identical conditions in both cohorts, and all histology content was taught only in lectures, it was not likely that the mode of learning had causal effects on student performance in the histology week 3 quiz (Table 1). For the remaining content,

- Face-to-face: Students were examined in a mid-semester practical examination (30% of total mark), and end-semester practical (30%) and theory (35%) examinations. All examinations were conducted similarly to examinations in the undergraduate face-to-face unit.
- Online: Students were examined in mid-semester (40% of total mark) and end-semester (55%) online examinations. Both examinations were conducted similarly to examinations in the undergraduate online unit.

**Table 1:** Descriptive data of undergraduate and postgraduate cohorts of students

	Undergraduate		Postgraduate	
	Online	Face-to-face	Online	Face-to-face
N total	86	60	71	67
N (% of total) mark	83 (97%)	55 (92%)	70 (99%)	65 (97%)
N (% of total) descriptive	75 (87%)	39 (65%)	11 (15%)	12 (18%)
Age (years)	18.8 (2.6)	19.0 (1.8)	24.1 (3.5)	22.7 (1.2)
Sex (M:F)	34:41	16:23	0:11	2:10
ATAR	78.9 (20.1)	87.8 (6.4)	88.0 (8.7)	88.3 (6.0)
Histology mark	–	–	9.1 (1.4)	8.9 (1.2)
ESE mark	44.4 (15.8)	52.7 (12.6)	80.5 (14.1)	72.4 (12.4)

ATAR: Australian Tertiary Admission Rank, ESE mark: end-semester examination mark, N total: total number of students enrolled in the cohort, N (% of total) mark: number of students with available end-semester examination marks, and histology online examination marks (postgraduate cohort only), N (% of total) descriptive: number of students with available descriptive data. Summary statistics of age, sex, ATARs, Histology and ESE marks were based on these sample sizes

### Outcome measures

Under the assumed causal graph, only the end-semester examination marks are to be compared between cohorts of students who learned by face-to-face or online modes. For students who learned by face-to-face mode, the combined end-semester practical and theory examination marks were analysed. For students who learned by online mode, the single end-semester online examination marks were analysed.

### Data processing and analysis

For each cohort in each unit, datasets of examination marks and descriptive data (age, sex, ATAR, course code) were linked using university student identifiers. Student identifiers were removed, and dummy identifiers were assigned. Analysis was performed on the cleaned datasets.

For each unit, end-semester examination marks were compared between face-to-face and online cohorts using linear regression. Between-group mean differences (95% CI) are reported. Data were processed using Python v3.9.

Estimates of the effect of face-to-face versus online learning on student performance could be biased by unmeasured confounding. For each comparison, we conducted sensitivity analyses to determine the ‘exposure-’ or E-value, the minimum strength of association (quantified as a risk ratio) that an unmeasured confounder would need to have with an exposure and an outcome to completely explain away an exposure-outcome association, conditional on any measured variables for adjustment [24, 25]. A large E-value indicates that substantial unmeasured confounding would be needed to explain away an effect. A small E-value indicates that little unmeasured confounding would be needed to explain away an effect. For example, an E-value of 2 indicates that unmeasured confounding would have to (1) double the probability of performing well in the examination among face-to-face or online students and (2) be twice as prevalent among face-to-face students than among online students, to completely “explain away” any observed effect, but weaker confounding could not explain away the effect [25]. Sensitivity analyses were conducted using the R-package Evalue [25].

All de-identified data, computer code, code to specify the causal graph, and study notes are available from the project repository (https://github.com/joannadiong/Diong_et_al_2022_DiscovEduc). We make available the study notes so investigators may see the thought processes behind how we developed and revised the causal graph.

## Results

Descriptive data of the undergraduate and postgraduate cohorts of students are shown (Table 1). End-semester examination marks were not available for all enrolled students because some students did not complete the units. Descriptive data were obtained centrally by the University, and were not available for all enrolled students.

**Fig. 2:**
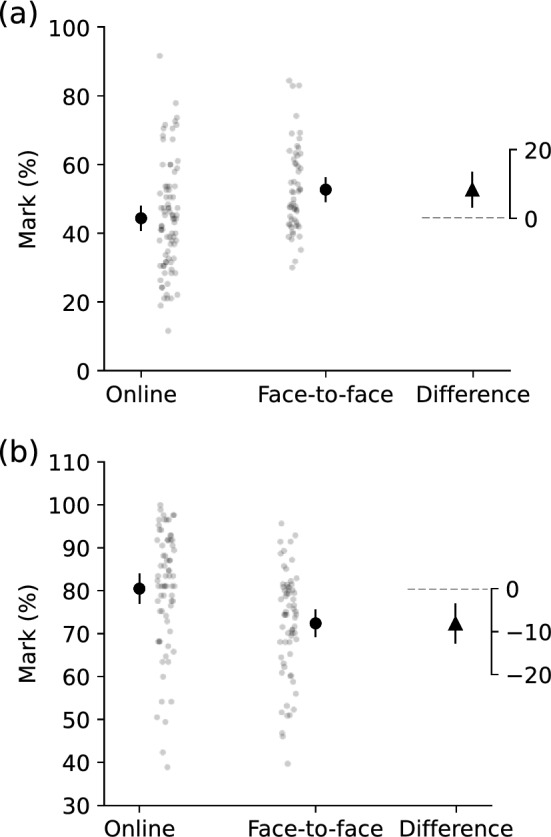
Within- and between-cohort descriptive and inferential statistics of end-semester examination marks of **A** undergraduate students and **B** postgraduate students. Within-cohort means and 95% CI (filled circles and error bars), individual participant data (grey dots), and between-cohort mean differences and 95% CI (filled triangle and error bars) about no difference of 0 (grey dashed line) are shown. Data were plotted using the Python package pliffy [29]

Compared to online learning, face-to-face learning improved student performance in the end-semester examination in undergraduate students (Fig. 2A), but not in postgraduate students (Fig. 2B). Undergraduate students who learned by face-to-face mode achieved a mean of 8.3% (95% CI 3.3 to 13.4%) more marks than students who learned by online mode. The E-value was 2.77 (lower limit of 95% CI 1.80). In contrast, postgraduate students who learned by face-to-face mode achieved 8.1% (95% CI 3.6 to 12.6%) fewer marks than students who learned by online mode. The E-value was 2.89 (lower limit of 95% CI 1.88). The E-value for the 95% CI only applies to the CI limit closer to the null risk ratio of 1 [25].

## Discussion

This study addresses the main limitation of many observational studies in this field by estimating the causal effect of face-to-face compared to online learning on student performance, under a causal structure. Under the assumed causal graph, we found that compared to online learning, face-to-face learning improved student performance in the end-semester examination in undergraduate students, but worsened student performance in postgraduate students. In both units, the mean improvement or decline of ~ 8% was reasonably large, and could vary from ~ 3 to 13% with 95% confidence.

These effects seem reasonably robust to unmeasured confounding. In the undergraduate unit, the E-value showed that there would have to be unmeasured confounding that improved student performance in either cohort by 2.77 times and was 2.77 times more prevalent in face-to-face than online mode students, above and beyond any measured confounders, to be sufficient to completely explain away the mean improvement of 8.3% marks in students who learned by face-to-face mode. In the postgraduate unit, the E-value showed that there would have to be unmeasured confounding that improved student performance in either cohort by 2.89 times and was 2.89 times more prevalent in face-to-face than online mode students, above and beyond any measured confounders, to be sufficient to completely explain away the mean decline of 8.1% marks in students who learned by face-to-face mode. These E-values seem large, and imply that substantial unmeasured confounding would be needed to explain away the observed effects. The lower limit of the 95% CI for both E-values still exceeds the null risk ratio of 1. Thus, the observed effects in both units are unlikely to be at high risk of bias from unmeasured confounding.

Under the assumed causal graph, the ATAR is not a common cause of the mode of learning and the end-semester examination mark. Therefore, no adjustment of exam marks by ATARs is indicated to determine the total effect of the mode of learning on student performance. In fact, improper adjustment of the between-cohort mean difference by ATARs would create a spurious association between the mode of learning and student performance, and bias the result [26].

Why did face-to-face learning improve undergraduate but not postgraduate student performance? Undergraduate and postgraduate students might have had different needs and styles of learning that predisposed them to performing differently under face-to-face or online modes [27, 28]. For example, undergraduate students might have needed teachers to demonstrate functional anatomy concepts in person, reinforced by using cadaver specimens and other media, in order to master the content. Undergraduate students might also have needed more structured learning in face-to-face classes, informal accountability, and physically learning in the social context of large and smaller groups to perform well [27]. In contrast, as postgraduate students tended to be older, they may have had more life experiences to be proficient at independent learning. The online mode could have afforded students greater flexibility for self-regulated learning [27] while balancing other responsibilities such as work, family commitments and childcare.

Our findings are consistent with some previous findings that showed face-to-face learning improved undergraduate occupational therapy student performance [6], but contrast with others that showed face-to-face learning worsened undergraduate dental student performance [7]. Likewise, our findings also contrast with other findings that showed graduate medical students made comparable knowledge gains in either mode of learning [8]. Above, we outlined mechanisms that might explain why student performance is different in undergraduate and postgraduate cohorts under different modes of learning. Importantly, our findings may be consistent or contrast with findings from other published research because, in many observational studies, the causal effects of the mode of learning on student performance were not assessed under a causal structure. Without a structural definition of confounding, causal estimates are prone to bias [9]. The key contribution our study makes is that it is the first attempt to estimate the causal effect of face-to-face learning on student performance in anatomy under a causal structure (Fig. 1). In doing so, we make our assumptions transparent before making our conclusions [9], and the causal structure guides how data are analysed (i.e. only the end-semester examination mark was analysed, not the total mark).

Accurate inferences of the causal effect of the mode of learning on student performance depend on a causal graph that is correctly specified using subject matter knowledge. Under the assumed causal graph, if the mid-semester examination mark and feedback were not sufficient to block causal effects from the *COVID lockdown* to *Student motivation* and *Teacher engagement* (Fig. 1), between-cohort differences may have been caused by these mechanisms other than the mode of learning. There could also be alternative mechanisms by which the COVID lockdown affected student performance that were not specified in the causal graph. For example, students who learned by online mode in 2020 were living under very different pandemic circumstances, compared to students who learned by face-to-face mode in 2019. The former cohort of students likely had to deal with concurrent physical, mental, social and economic challenges (e.g. minimising exposures to COVID, family or household stressors, potential unemployment, etc.). A limitation of this study is that our causal graph assumes these factors did not contribute to the overall causal effect. Or, if they did, their effects were captured within *Student motivation*, and we assumed the mid-semester examination mark and feedback were sufficient to block causal effects through this path. If future investigators could improve on our causal graph to find ways to adjust for or block alternative causal effects, it may be possible to obtain more robust estimates of the effect of the mode of learning on student performance. More broadly, assessing the overall effect of the pandemic on societal outcomes and its academic, economic, social and emotional costs will likely provide valuable information to educators and higher education institutions in navigating a way forward in these unprecedented times [19].

In summary, compared to online learning, face-to-face learning improved student performance in undergraduate students but not in postgraduate students. The average improvement or decline in performance was reasonably large, was estimated with precision, and was reasonably robust to unmeasured confounding. Importantly, this is the first attempt to estimate causal effects of the mode of learning on student performance under a causal structure. This makes our assumptions transparent, and guides how data are analysed. In future, investigators could extend on our causal graph to obtain more robust estimates of causal effects, or assess broader overall effects of the pandemic on learning and education.

## Data Availability

All de-identified data, computer code, code to specify the causal graph, and study notes are available from the project repository (https://github.com/joannadiong/Diong_et_al_2022_DiscovEduc).
